# Provisional Tic Disorder: What to tell parents when their child first starts ticcing

**DOI:** 10.12688/f1000research.8428.1

**Published:** 2016-04-18

**Authors:** Kevin J Black, Elizabeth Rose Black, Deanna J. Greene, Bradley L. Schlaggar

**Affiliations:** 1 Departments of Psychiatry, Washington University School of Medicine, St. Louis, USA; 2 Departments of Neurology, Washington University School of Medicine, St. Louis, USA; 3 Departments of Radiology, Washington University School of Medicine, St. Louis, USA; 4 Departments of Neuroscience, Washington University School of Medicine, St. Louis, USA; 5 Brigham Young University, Utah, USA; 6 Departments of Pediatrics, Washington University School of Medicine, St. Louis, USA

**Keywords:** Provisional Tic Disorder, Tourette syndrome, tic remission, tics

## Abstract

The child with recent onset of tics is a common patient in a pediatrics or child neurology practice. If the child’s first tic was less than a year in the past, the diagnosis is usually Provisional Tic Disorder (PTD). Published reviews by experts reveal substantial consensus on prognosis in this situation: the tics will almost always disappear in a few months, having remained mild while they lasted. Surprisingly, however, the sparse existing data may not support these opinions.

PTD may have just as much importance for science as for clinical care. It provides an opportunity to prospectively observe the spontaneous remission of tics. Such prospective studies may aid identification of genes or biomarkers specifically associated with remission rather than onset of tics. A better understanding of tic remission may also suggest novel treatment strategies for Tourette syndrome, or may lead to secondary prevention of tic disorders.

This review summarizes the limited existing data on the epidemiology, phenomenology, and outcome of PTD, highlights areas in which prospective study is sorely needed, and proposes that tic disorders may completely remit much less often than is generally believed.

## Introduction

*Most parents want predictions of the future for their child. This, however, is one of the more challenging aspects of caring for the child with [tics]* ^1^.

Pediatricians, child neurologists and child psychiatrists commonly encounter children with recent-onset tics. Many experts conclude that Provisional Tic Disorder (PTD)—tics in someone whose first tic was less than a year ago—is probably a different disease than chronic tic disorders including Gilles de la Tourette syndrome ^1– 7^. Others conclude that all tic disorders lie on a continuum, with no appropriate arbitrary duration boundary, and may share the same causes ^8– 11^. However, most experts agree that current PTD is usually mild and will likely go away in a few months ^1– 5, 12– 16 (p. 171)^. Surprisingly, the sparse existing prospective data may not support this prognosis. This article reviews the current state of knowledge about PTD, focusing on clinical relevance.

## Definitions

Tics are abnormal, unwanted movements or vocalizations. They are distinguished from other movement disorders by several characteristics: they are repeated, stereotyped, discrete and nonrhythmic, most frequently involve the head and upper body, and (although sometimes described as involuntary) are perceived by most patients as an inevitable capitulation to an almost irresistible urge ^17^. Typical examples of tics include raising the eyebrows, turning the eyes, shaking the head, sniffing, snorting, and more complex phenomena such as repetitive touching or saying words or phrases ^18^. Tics almost always begin in childhood, about ages 3 to 9 years ^19– 21^, and on average tics are most severe around ages 9 to 11 ^22^. Recent-onset tics caused by a systemic or other neurological illness are not considered further in this review, since most tic disorders are primary ^23^.

A brief digression into nosology is necessary to understand the primary literature. Differences between diagnostic criteria contribute to confusion about the patient with recent-onset tics, since various criteria sets differ on defining features, such as whether a minimum duration of ticcing is needed for diagnosis, or whether brief reappearance of tics after a remission of months to years confirm a single chronic disorder or constitute a second episode of transient tics. Similarly, several diagnostic schemata defined “transient tic disorder” (TTD), but construed the term “transient” in quite different senses. These points complicate interpretation of the literature, because many published reports used different rules than those they claimed to use. Appendix 1 discusses these points in more detail. The simplest definition in current use comes from DSM-5, which specifies that a child with primary tics that began less than a year ago is always diagnosed with Provisional Tic Disorder ^12, 24^. We will follow that nomenclature when referring generally to the child with recent-onset tics, but when reviewing the primary literature we attempt to specify the rules the various reports actually used for definition.

The common, idiopathic DSM-5 chronic tic disorders are Tourette’s Disorder (also called Tourette Syndrome) and Persistent (also called chronic) Motor (or Vocal) Tic Disorder. Hereinafter the terms Tourette Syndrome or Chronic Tic Disorders (TS/CTD) are used.

## Search strategy and selection criteria

References for this review were identified by searches of PubMed through February, 2016, and references from relevant articles and books. Search terms included “tic disorder”, “transient tic disorder”, “Tourette NOT Tourette [AU]”, and “tic disorder [MAJR] AND (transient [TW] OR persistent [TW])”. The final reference list was generated based on relevance to the topic of this review.

## Epidemiology of Provisional Tic Disorder

*Tics are thought to be the most common movement disorder diagnosed in children* ^25^.

### Prevalence

Estimates of the prevalence of PTD span a wide range ^25– 30^. For example, at least one motor tic was observed in 47% of first graders over the course of one school year ^31^, while a study using questionnaires and interviews reported a 5% lifetime prevalence of PTD (the authors reported 2.6% as DSM-IV TTD, plus 2.5% in a separate category for children with current tics that had begun < 12 months prior) ^32^. Obviously at least one of these estimates must be wrong. Below we discuss the key issues in epidemiological studies of PTD before returning to a summary of the evidence.

### Factors that contribute to the wide range of prevalence estimates.

A number of factors complicate estimation of PTD prevalence, and some of these deserve special attention. A more complete list and additional references appear in Appendix 2.

***Point prevalence vs. lifetime prevalence.*** Cross-sectional studies are much easier to conduct than longitudinal studies, but provide very different information. Nevertheless, all prevalence studies of chronic tic disorders are relevant to the lifetime prevalence of PTD, because all old tics were once new. In other words, all children with TS or CTD started ticcing at least a year ago, and in the first few months after tic onset would have met criteria for PTD. So the total number of children with a lifetime diagnosis of *any* tic disorder constitutes a lower bound for the lifetime prevalence of PTD.

Furthermore, since by definition PTD has not lasted for a year, resampling even a short time later can substantially change the prevalence estimate. The most illustrative data come from Snider and colleagues ^31^, who visited an elementary school eight times over the course of a single school year. A trained rater observed the classroom for 1 hour on each visit, attending to each child individually for at least 3 minutes. A motor tic was observed at some point during the year in 47% of first graders [Table 1 in ref. 31]. However, the children in first grade in one year will be in second or third grade in subsequent years, and observers saw tics in children in all grades. One can calculate from their data that by sixth grade, at least 70% of students must have met the DSM-5 criteria for PTD at some point (for details, see Appendix 3).

***Population.*** Prevalence of tic disorders depends heavily on the age of the population studied. A prospective, community-based study found that “the percentage of children with tic behaviors varied with age: preschool children (22.3%), elementary school children (7.8%), and adolescents (3.4%)” ^33^. Similarly, the 1-year period prevalence of tics with impairment or distress was 9% in children age 7–9, 6% age 10–12, and 5% age 13–15 ^34^. In another grade school study, the prevalence of directly observed motor tics peaked in first grade (~age 6) ^31^. By contrast, a large study identified TS in only 12 of 28,037 teenagers screened at age 16–17 ^35^.

Tics are much more common in children with intellectual disability compared to healthy children without developmental delay, and in special education classrooms than in regular classrooms ^33, 36– 39^. In Khalifa and von Knorring’s study, for instance, the 1-year prevalence of any DSM-IV tic disorder was 6.3% in children in regular classrooms but 46.3% in special school settings ^34^. Tics are also common in children with an autism spectrum diagnosis ^40^. Consequently, studies will underestimate prevalence if they do not take care to include children outside mainstream classrooms.

***Sampling method***

*Generally speaking, few patients with tics seek medical advice, since tics are thought to remit spontaneously* ^41^.

The classroom setting is a special case; more generally, any convenience sample is likely not to fairly represent the entire population. Specifically, children evaluated in any health care setting are more likely to have experienced severe tics and to have symptoms other than tics ^42^. In studies that use a staged approach to sample a population, an important concern is the false negative rate of the screening procedure. If, for example, 5% of the original population screen positive, then even if the true negative rate of the screening procedure is 90%, the study will underestimate the true prevalence by about two thirds. In perhaps the most telling example, eight screen-negative children—no tics reported by parent, teacher or self, and no tics observed by an expert visiting the classroom—came in for a 20-minute interview, and tics were observed in three of the eight ^42a^.

***Sources of information.*** Several studies show that direct examination of children identifies tics that children, parents and teachers were all unaware of. This result is apparently due to true differences between sources rather than poor within-source reliability: even though “rates of motor tic frequency were found to be moderately stable across both days and school settings,” clinician ratings of tics correlated only moderately with tics observed by researchers or by teachers ^43^. Historical information is also crucial, because tics can be absent during an examination due to spontaneous fluctuations in severity, tic suppression, or effective treatment. In general, studies that employ a more comprehensive approach to identifying cases find a higher prevalence of tics ^25, 44– 46^.

***Prevalence: conclusion.*** In summary, many factors complicate accurate estimation of tic prevalence, and most of these lead to underestimation ( Appendix 2). At a given moment in time, the fraction of children with either chronic or recent-onset tics constitutes a lower bound on the lifetime prevalence of PTD. Point prevalence depends strongly on age, with the highest rate probably about 20% at age 5–10. Lifetime prevalence is much higher; though longitudinal studies have been sparse, the available evidence supports the view that tics occur at some time or another in a large fraction of all children, probably over half. In most cases these tics never come to medical attention, and those that do are disproportionately more severe. Tics are even more common in children with attention deficit hyperactivity disorder (ADHD), obsessive compulsive disorder (OCD) or intellectual disability.

### Incidence

*It is almost impossible to conduct a prospective study of the onset of tics, as the majority of patients do not seek help for the tics but rather for other problems* ^47^.

Incidence means the rate of new (first-episode) cases in a given period of time. Some of the prevalence studies cited above provide limited information on incidence. Spencer and colleagues ^48^ provide some of the most direct data, prospectively observing boys for 4 years. The rate of new-onset tic disorders over the 4-year follow-up period was 3% in boys without ADHD.

Spencer *et al.* also provide incidence data in boys with ADHD, many of whom had tics at baseline (17%, vs. 4% in boys without ADHD). Boys with ADHD were more likely than those without ADHD to develop new tic disorders over the 4-year follow-up period (20%, including 33% in the 6- to 8-year-old group). Law and Schachar ^49^ performed a large, 1-year-long randomized controlled trial of methylphenidate for ADHD, and monitored carefully for tics. Children who met the criteria for TS at baseline were excluded from participation, but (other) tics were common at baseline (30%). During the year of follow-up, “clinically significant tics for the first time ( *i.e.*, moderate or worse)” occurred in 19.0% (12/63) of the children who had no tics at baseline. The relevant part of this study for this section is the high rate of new tic disorder in the *placebo* group: one sixth (16.7%, 2/12) of children with ADHD but no tics at baseline developed PTD of at least moderate severity in one year (see also Table 2 in ref. 49).

Carter and colleagues ^50^ prospectively followed first-degree relatives of TS probands for 2 to 4 years. Nine of 21 children free of tics initially but at least 4 years old at final assessment had developed tics: five were diagnosed with TS, two with chronic motor or vocal tic disorder, and two with DSM-III-R TTD (one of whom developed tics just before the last assessment). In other words, at least one third of children in this high-risk sample developed a chronic tic disorder over an average of only 3 years. In a similar study, nine of 29 children of a parent with TS had a tic disorder at baseline, and 10 more developed a new tic disorder over the next 2–5 years ^51^.

## Clinical features of Provisional Tic Disorder

Given its prevalence, the knowledge base on PTD is surprisingly limited and scattered ^9, 47^. Some authors report that PTD that remits within the first year has a similar initial presentation as do chronic tic disorders ^2, 52, 53^. Certainly any clinical feature of TS can present first chronologically (when the diagnosis is still PTD). However, some features of TS are less typical in PTD: not all children with PTD progress to TS, and some features of TS are more common in older children. Many of the conclusions below should be taken as tentative, since cross-sectional studies *not* focused on recent tics supply most of the data.

### Demographics

Boys are more likely to have PTD than girls, with reported sex ratios of 1.2–4.0 ^31, 41, 54– 56^. The sex ratio in TS is generally reported as closer to 4. In this regard it may be relevant that children with tics spanning at least 3 months had a significantly higher ratio of boys to girls (7.5:1) than did the children with tics observed in only 1 month or 2 consecutive months (1.6:1) ^31^; perhaps tics persist more often in boys.

In a large epidemiological study, children with DSM-IV TTD had a later age of onset than children with TS/CTD ^47^. This finding is plausible, but a longitudinal study is required since chronicity is probably easier to recall in children with more severe or less socially acceptable tics ^34^.

### Tics and other symptoms

Children with DSM-IV TTD had lower severity and a lower rate of vocal tics than children with TS or CTD ^47^. Similarly, in a school observation study, children with tics spanning at least 3 months had a significantly higher mean tic severity than children with tics observed in only 1 month or 2 consecutive months ^31^. In a report on over 300 children with ADHD, tic disorders (chronic and recent-onset together) were two to three times as common in combined type ADHD than in hyperactive-impulsive or inattentive ADHD ^57^. Case-control and family studies of OCD find an excess of tic disorders in OCD relatives, and suggest that tics are more common and more severe in relatives of boys with OCD beginning earlier in childhood ^58, 59^.

### Treatment response

Treatment response in PTD has not been carefully studied, in part because treatment is often unnecessary. Clinical experience suggests that symptoms in PTD respond to treatment at least as well as in TS/CTD.

## Etiology

### Genetics

In 1987, Zausmer and Dewey opined that “Further research is needed to confirm that there is a connection between childhood tics and Gilles de la Tourette's syndrome, to establish that the predisposition to tics is familial, and, if so, whether there is a complex genetic mechanism involved, or some other environmental ætiology so far undisclosed” ^60^. However, the following year Kurlan *et al.* reported on two individuals with remitted PTD in TS families whom they concluded were “very likely obligate carriers of the TS gene” ^61^, and in fact most family studies of probands with TS find relatives with other tic disorders ** including PTD. Of 16 monozygotic twin probands with TS, 56% of co-twins also had TS, but 94% of co-twins had some tic disorder, suggesting strongly that TS and other tic disorders share a genetic predisposition ^62^. High rates of tic disorders including PTD in probands and family members of patients with ADHD and OCD have suggested that these clinical features may be “alternative manifestations of the same underlying illness” ^7, 51, 59, 63^.

### Environmental factors

In the monozygotic twin study of TS cited in the previous paragraph, the lower birth-weight twin had more severe tics in 12 of 13 pairs, and “the magnitude of the intrapair birth-weight difference … strongly predicted the magnitude of the intrapair tic score difference” ^62^. These results suggest that environmental factors *in utero* may predict severity and outcome of tic disorders. Maternal smoking or other drug use during pregnancy may also be risk factors for TS ^64– 67^ and thus perhaps also for PTD. Although external contingencies do transiently modify ticcing in PTD ^68^, life events seem to have little to do with the onset of TS/CTD ^69^. Life events have not been evaluated systematically in PTD.

## Outcome of PTD

*Studies of prognosis are infrequent and difficult to conduct, and the conclusions are severely limited by diverse methodological shortcomings [ 16, p. 188].*

One of the most important questions about PTD remains that of prognosis. Since prospective studies of PTD are so few, we examine cross-sectional and retrospective studies as well.

### Outcome inferred from cross-sectional studies

Child epidemiological studies not focused primarily on tic disorders often identify too few cases to be useful for this context ^70, 71^.

***Age and sex.*** Subjects with DSM-IV TTD later recalled their age of onset as 8.5 ± 3.0 years, *vs.* 4.6 ± 2.9 for TS, though onset for chronic motor or vocal tics also averaged 7–8 years, and a prospective study would be needed to rule out recall bias ^47^. In Snider *et al.*’s grade school observational study, the boy:girl ratio was higher in “the persistent group,” *i.e.* tics observed on at least three consecutive monthly visits or two nonconsecutive visits (7.5:1), than in the isolated group, *i.e.* tics observed only on one visit or two consecutive visits (1.6:1) ^31^.

***Tics and other symptoms.*** In the Snider *et al.* “persistent group,” non-facial tics were twice as common as in the isolated group, and problem behaviors were also more associated with tics in the persistent group ^31^. In a large epidemiological study, TTD subjects had lower tic severity than children with TS/CTD, and were less likely to have vocal tics ^47^. In the same study, people with vocal tics (at least chronic vocal tics) had higher rates of comorbidity (58% vs. 12%), including ADHD (33% vs. 12%) and OCD (8% vs. 0%).

***Duration at initial presentation.*** Editions of the Diagnostic and Statistical Manual previous to DSM-5 had required a 4-week threshold for diagnosing TTD, but a recent expert consensus statement noted no basis for this threshold in data, concluding that “it is unknown whether tics of less than one month’s duration predicts a transient course or not” ^24^. In the Snider *et al.* “persistent group,” tics were significantly more severe than in the isolated group (mean 1.08, t = 2.7; P < .01)” ^31^.

### Outcome based on retrospective data

Remschmidt and Remschmidt located 54 families of children who had been seen for tic disorders ^72^. After an average of 3 years, 11 (38%) of the 29 untreated children had completely remitted according to the family. Abe and Oda ^73^ obtained questionnaire data from the parents of 32 of 57 children of a parent with tics, and 94 of 178 control children, chosen from children seen at a well-child clinic when they were 3. Of the children in the former, high-risk group, 25% were reported to have tics at age 8, compared to 10% of the control children. Stárková reported the duration of clinical treatment in 131 tic patients hospitalized in a Czech child psychiatry department over a 20-year period ^74^. Duration of tics at onset is not provided, so many of these patients may have had TS/CTD at initial presentation, but 38% of the 63 patients aged 15–29 years at last follow-up had completely remitted.

***Age and sex.*** At a one-day clinic in New York in 1977, members of 21 families with a GTS proband were examined. Many family members had current or past tics, and “among those with spontaneous clearing, females predominated” ^75^.

***Tics and other symptoms.*** Wang and Kuo concluded that: “Cases in 4–6 years old children with multiple motor and vocal tics have poor prognosis” ^76^. Consistent with that opinion, six of 11 spontaneously remitting tic disorders in another study had only a winking tic at presentation, and only one had a vocal tic ^72^. By contrast, Chouinard and Ford concluded that “the appearance of the tic disorder, the course and prognosis, the family history of tic disorder, and the prevalence of OCD were found to be similar” in nine adult patients in whom a previous history of transient tics in childhood was eventually elicited, whereas a specific cause for the tic disorder (infection, trauma, cocaine use, or neuroleptic exposure) was more often found in 13 adult patients who apparently were truly presenting *de novo* ^53^. In a large study of adults, “a significantly greater proportion of adults with ADHD (12%) than those without ADHD (4%) had tic disorders. Tic disorders followed a mostly remitting course and had little impact on functional capacities” ^77^.

***Remission at presentation.*** In the epidemiological study of Wang and Kuo, all subjects diagnosed with TTD in childhood had remained in remission ^76^. However, this conclusion, like so many discussed in the preceding sections, may depend on the thoroughness of the assessment. “Detailed questioning disclosed a history of previous childhood transient tic disorder” in nine of 22 patients presenting for medical care of a tic disorder for the first time after the age of 21 ^53^. Similarly, children with tic disorders that had remitted before age 20 often experienced a recurrence later in life ^78^. Still, the remission rate for PTD is likely more favorable than that of TS; tics persisted into adulthood in 90% ^79^ or 100% ^80^ of patients with childhood-onset TS.

### Outcome from prospective monitoring

*Statistics on the rate of permanent spontaneous remission are difficult to obtain since these patients, characteristically, do not return for follow-up contacts ^81^.*

The ideal design to address prognosis of PTD is a prospective study, lasting at least through the 1-year anniversary of the first tic. Unfortunately, few such studies are available. Shin and colleagues followed eight children (seven boys) with DSM-III or DSM-III-R TTD ^82^ identified by hospital records. Their age at symptom onset was 8.38 ± 3.60 years (mean ± SD), and symptom duration at presentation was 0.38 ± 0.37 years. At follow-up 3 to 18 years later, using a semi-structured interview by telephone or face to face, four had recovered completely and four had not improved at all. By comparison, of the 22 children in the same study who were initially diagnosed with TS or CTD, seven had remitted by follow-up, 11 had improved somewhat, and four had not improved.

Bruun and Budman reported follow-up by telephone (62%) or direct interview (38%) on 58 children who had tics lasting less than a year at presentation (DSM-III and ICD9 TTD) ^9^. After 2–14 years, tics had remained absent throughout the follow-up period in only 17%; 40% now met criteria for chronic motor or vocal tics and the remaining 43% “continued to have tics that were chronic and episodic (either TS or tic disorder ‘not otherwise specified’ by DSM-IV criteria)” ^9^.

Peterson *et al.* ^83^ followed for up to 15 years a large sample of children with chronic or recent-onset tics, diagnosed initially by parental report only. The follow-ups used direct examination for diagnosis. The number of children with motor tics declined substantially over time (Time 1, 17.7%; Time 2, 2.2%; Time 3, 2.1%; Time 4, 0.6%), but as only Times 2 and 3 used identical ascertainment methods, generalization about remission of PTD is difficult. From 43% to 84% of 32 children with a tic at presentation to an ophthalmology practice still had tics after an average follow-up of 6.1 ± 3.9 years ^84^ (diagnosis at presentation was not reported, but many of these children probably had PTD). A few other studies report limited prospective data ^52, 85, 86^.

***Age and sex.*** Corbett *et al.* ^56^ found prospective follow-up data after a mean of 5.4 years on 73 of 89 children who had first seen the doctor specifically because of tics. These 89 were part of a larger sample of 180 children with tics. Of the 122 subjects with adequate information recorded about the date of tic onset, at least 44 had experienced tics for more than a year at the initial assessment. Thus many of the 73 children with prospective follow-up data probably had PTD at the baseline visit, but many already had chronic tics. With this caveat, one interesting point is that outcome depended on age of onset; remission was more likely (16 of 26, 62%) in children whose tics had started at age 6–8 years than in children whose tics began at age 2–5 (7 of 29, 24%) or at age 9–15 (6 of 17, 35%; p<.02; Table VIII in 56). In the same study, full remission was significantly more likely the longer the follow-up, so that only 32% of those followed for 2 years or less were tic-free, *vs.* 65% of those followed for at least 8 years. In other words, many children who do remit completely will do so only after meeting criteria for TS or CTD.

Spontaneous remission was more likely in girls than boys in one follow-up study of mixed tic disorders ^87^, but a similar study found no relationship of remission to sex ^88^. The latter study also suggested better prognosis for children whose parents had tics that remitted vs. persisted in adulthood.

***Tics and other clinical features.*** In a clinical sample of 26 children initially diagnosed with TTD, coprolalia during the first year did not differentiate those later diagnosed with Tourette’s disorder from those still diagnosed with TTD at follow-up 1–11 years later [ 16, pp. 373–374]. In the same study, none of the three children who presented with a complex tic remitted, though the group difference was not significant. However, “children were more likely to develop Tourette's disorder if they had three or more vocal symptoms during the first year (8 [of] 17 children); all of the children with less than three vocal symptoms had a final diagnosis of TTD (p = 0.02)” [ 16, p. 374]. Bruun and Budman also reported significantly better prognosis in children who presented without vocal tics ^9^.

None of the children who remained in remission had a tic below the neck at their first visit, compared with at least 19% of those later diagnosed with TS [ 16, pp. 373–374]. In fact, all four children who had a lower extremity tic at any point in the first year were diagnosed with TS at follow-up. Neither of these results was statistically significant, but the mixed tic disorder follow-up of Corbett *et al.* similarly found a trend (p<0.10) for fewer remissions in patients with a lower extremity tic at presentation ^56^.

In the prospective study by Peterson *et al.*, ADHD at baseline was associated with later tic persistence ^89^. Spencer *et al.* prospectively monitored the appearance and disappearance of tic disorders over the course of 4 years in boys with or without ADHD who did not have TS at study entry ^48^. Almost all (~90%) of the boys with tics had a chronic tic disorder, so the data are only partially relevant. Still, over the course of 4 years, the age-adjusted rate of remission was 65% for tic disorders (vs. 20% for ADHD).

One study located 54 children with OCD who had enrolled in OCD treatment studies for which Tourette’s disorder had been an exclusionary criterion, and re-examined them “2–7 years later with a neurological examination and a structured interview to establish the presence or absence of tics and Tourette's disorder” ^59^. At baseline, 16 of the 54 children had a current tic disorder, and 15 other children had a past history of tics. Twelve of the 31 had a diagnosis of DSM-III-R TTD at baseline. At follow-up, 17 of the 31 had current tics, three others had a past diagnosis of TS/CTD, and for 12 the only lifetime tic diagnosis was TTD. One additional person with no tics at baseline had developed a tic disorder (Tourette’s). Thus in this study of childhood OCD, most children had current (30%) or past (28%) tics at baseline, though none were thought to have TS. At follow-up, 31% had a current, chronic tic disorder, but those with a previous diagnosis of TTD had remained remitted. The study was focused on tics only at follow-up, but bearing that caveat in mind, the results suggest the possibility that OCD at baseline may protect children whose tics have already disappeared from tic recurrence over the next few years.

Unpublished data suggest that among children with tics for less than 6 months at study entry, those who could suppress tics better when asked to do so had more improvement in tic severity at the 12-month anniversary of tic onset ^90^.

### Outcome of PTD: summary

A few indisputable facts are evident from the existing data on outcome of PTD. Some children who start ticcing will go on to have chronic tics, often associated with impaired quality of life. (Still, prognosis even for TS is primarily hopeful: in a group of children with TS, 11.4 ± 1.6 years old, one third had a YGTSS score of 0 when followed up a mean of 7.5 years later; *i.e.*, no evidence of tics over the past week.) At the other extreme, tics will remit completely in many children with recent-onset tics—or, more likely, will cease to be noticed or to affect quality of life.

However, the existing data provide little certainty about outcome even collectively, much less for individuals. The best-quality information we have comes from the few studies limited to patients identified during the first year of ticcing that either followed the patients prospectively or achieved good follow-up rates retrospectively (see Table 1). Overall, these studies suggest that a child with tics who had no tics prior to 1 year ago has a favorable prognosis overall, but has only about a one in three chance of remaining completely tic-free over the next 5–10 years. Many of those who do remit probably remit only after meeting criteria for TS/CTD at some point.

**Table 1.** Prospective outcome studies of PTD.

Citation	*N*	Duration	Remission
Shin 1996 ^82^	4	3–18 yr	50%
Shapiro 1988 [ 16, pp. 371–374]	26	1–11 yr	35%
Spencer 2001 ^77^	36	1–55 yr	53%
Bruun and Budman 1997 ^9^	58	2–14 yr	17%
**TOTAL**	**124**	**--**	**32%**

## Discussion

Overall, the existing data on PTD are relatively limited. Some of the reasons for this state of affairs are inherent in the problem, and others can be attributed to changing knowledge about tic disorders, diagnostic criteria that have changed several times over the past few decades, and an understandable preference for research to focus on patients with severe and persistent symptoms. Nearly every section above included important unanswered questions, but some of the most important include the following. Do most children, or only a minority, experience tics at some time during childhood? Are the causes of tic disappearance different from the causes of tic appearance? Why do tics usually go away—or at least cease to be a clinical problem? And not least, the question we started with: Will my child’s tics go away, or get worse?

### Implications for nosology

The conclusion that complete and permanent remission of recent-onset tics may be relatively uncommon is consistent with a number of observations. First, some studies provide direct support for intermittent tics ^31, 46, 91^. In discussing the DSM-IV-TR criteria, Singer wrote: “Since children are permitted to have recurrent episodes of ‘transient tics’ and recognizing that tics may go unnoticed, this author would suggest that some individuals in this category actually have a CTD” ^92^. Second, the more comprehensive the assessment strategy, the higher are the rates of tics observed in epidemiological studies. To give one example, in a random population sample with very thorough clinical ascertainment, tics were observed in one sixth of children in mainstream schools ^38^ (see also Appendix 2). Cubo provides even more direct evidence for this effect [Table 2 in ref. 25]. Therefore, apparent remission of tics is likely to be much more common than complete remission. Third, many adults with tics are unaware of their tics ^9, 79, 80^, a fact acknowledged explicitly by the working group for DSM-5 as a reason for changing the diagnostic criteria so that any tics more than a year apart could be diagnosed as a chronic tic disorder.

Bruun and Budman suggest that a fluctuating course commonly follows TS: “It is the impression of these authors, rather [than complete, lifelong remission] that the more common course is one of occasional recurrences of mild tics throughout adult life” ^9^. Shapiro *et al.* provide prospective data on this point: “27.1% of our [TS] patients had one or more periods of spontaneous remission lasting from less than 1 month to 19 years” [ 16, p. 188]. Similarly, 62% of tic patients hospitalized as children, when followed up between the ages of 15 and 29, reported only occasional short-term tic relapses lasting several days and requiring no treatment; only 14% were considered to have a poor outcome ^74^. In discussing prognosis of TS/CTD, Singer concluded that “whether tics actually disappear completely is unclear, and results appear to be dependent on the methods used to document the presence of tics.” ^93^. The data reviewed above suggest that the same conclusion may apply to tics that began only recently.

The conclusion that the most common outcome of PTD is improvement without remission is true only if by remission one means zero tics ever again, consistent with DSM-5. However, this threshold brings with it some uncomfortable conclusions. Consider a patient who first developed winking and sniffing tics 13 months ago. He and his mother report that his tics have been gone for the past 6 months, and he shows no tics during a 45-minute office visit, but some of his old tics are observed when he sits alone for a few minutes. His DSM-5 diagnosis is Tourette syndrome even though for all practical clinical purposes he has remitted. This child is a substantial departure from the iconic (if unrepresentative) Marquess of Dampierre ^94^. Martino and colleagues address this problem using an idiosyncratic but understandable nomenclature, diagnosing “physiological tics” if the severity does not warrant diagnosing a “disorder.” They conclude that “‘physiological tics’ commonly occur during normal childhood development and reﬂect a stage of the physiological synaptogenesis within connections between basal ganglia and frontal lobes” [ 15 (pp. 105–107)].

A different solution is to reconsider the DSM-IV-TR choice to remove the “impairment or marked distress” criterion. The experience of Coffey and colleagues supports this view: “Although tics followed a persistent course in the majority of youth with TD [Tourette’s Disorder], they were infrequently associated with impairment. There was a significant reduction in the proportion of youth with TD impairment from baseline to follow-up. These results support the view that TD is a persistent disorder, but suggest a dissociation between tic persistence and tic-associated dysfunction” ^95^. However, for both biological and societal reasons, psychosocial consequences of illness, like “distress or impairment,” seem unsatisfactory in defining a highly heritable syndrome. Objective measures focused only on tic severity would sidestep these concerns, but valid, objective tic severity measures encompassing a period longer than a single office visit have been difficult to implement. In either case—whether one prefers to measure tics’ severity or their impact—a zero threshold inevitably produces the nosological frustration discussed in the previous paragraph.

### Implications for clinical care and research

***Prognosis***

*We do not know the cause of or have the ability to predict spontaneous waxing, waning, fluctuation, and temporary or permanent remission of symptoms* [ 16, p. 175].

Research to clarify the expected course of PTD would be greatly appreciated by the children and families who seek consultation for recent-onset tics. Firmer group estimates of improvement and remission rates from prospective studies would be welcome, but even more useful would be identification of additional features at presentation that help predict outcome on an individual level.

***Treatment***

*Transient tic disorder “is a self-limiting disorder, and active treatment typically is not indicated.” However, given limited follow-up data, “a child with a diagnosis of TTD should be periodically monitored and the diagnosis and treatment revised as necessary.”* ^2^

At the present time, experts generally agree that treatment for PTD is warranted only when symptoms are severe and persistent enough to substantially distress the child or interfere with his or her school experience or social development. However, better prognostic ability would allow the possibility of early intervention to improve the long-term outcome.

***Prevention***

*If we can … detect the brain changes years before the behavior starts, then there’s the opportunity to intervene early. And that’s where we do best in medicine. Early intervention, preempting the later stages, is where we’ve had our greatest successes. … At that point, we’ll start to see … the really big public health impact.* ^96^

*An ounce of prevention is worth a pound of cure. (Benjamin Franklin)*

Since Tourette syndrome and persistent (chronic) motor/vocal tic disorder (TS/CTD) are defined as having lasted at least a year from onset to most recent symptoms, one can envision that an effective intervention, supplied within months of the initial onset of tics, could conceivably prevent TS/CTD. No such intervention has been proven, but it is now clear that a behavior therapy approach (Comprehensive Behavioral Interventions for Tics, or CBIT) is reasonably effective and definitely safe for TS/CTD. If CBIT can be shown to improve the outcome of recent-onset tic disorders, it can become an approach to primary prevention of TS/CTD (secondary prevention of tic disorders). At present, factors such as cost and limited availability mean that treating all children with recent-onset tics is impractical, not to mention unnecessarily intrusive for the majority of children whose tics will disappear (or become mild or rare). However, those limitations could be overcome with better prognostic accuracy.

***Tics that go away can tell us something important about tics that don’t***

*Francis Bacon … pronounced that it must be of the greatest interest for the physician to study healed cases of incurable diseases* ^97^.

As reviewed above, very little is known about predicting outcome from studies of PTD itself. However, more follow-up data are available after tics have become chronic ^22, 79, 80, 95, 98– 106^. These follow-up studies of chronic tic disorders suggest hypotheses that can be tested in prospective follow-up studies of PTD. Leckman ^107^ lists the following potential prognostic features for TS: visual-motor integration ^108^, decreased TMS motor inhibition, ^109, 110^, MRI volumetry ^89^, and white matter integrity ^111^. Singer ^92^ adds: “Proposed predictors of severity and longevity include tic severity, fine motor control, … size of caudate and subgenual volumes ^99, 100^, but all are controversial ^22, 93^.” Here, “fine motor control” may refer to Purdue Pegboard performance ^112^. Additional potential markers for outcome of PTD include baseline ability to suppress tics ^68, 113^, resting state functional connectivity ^114^, probabilistic learning ^115^, ADHD at diagnosis ^116^, vocal tics ^9^ or tics below the neck at presentation, or more than two vocal tics in the first year [ 16, pp. 373–374], socioeconomic status (ref. 16, p. 175) and family history of TS/CTD, perhaps especially family history of tics persisting into adulthood ^88^ or tics in both parents ^51^.

A careful, prospective study that includes some of the most promising of these potential markers may allow discovery of the cause and pathophysiology of tic improvement, with the potential consequences for clinical care noted in the preceding section. This hope provides one of the key motivations for attempting to identify clinical, neuropsychological, or brain imaging variables that correspond to improvement *vs.* worsening of recent-onset tic disorders. As we tell parents who enroll their children in our ongoing study of recent-onset tics, “We’d love to find out what happens in the brains of kids whose tics improve spontaneously, and put it in a bottle or find out how to teach it to patients with chronic tics.”

## Figures and Tables

**Appendix 2.** Factors that complicate assessment of recent-onset tics The interested reader is also referred to insightful discussions of this topic by Cubo ^25^ and by Robertson and colleagues ^26^.

Problems	Comments and references
Children (and adults) don’t notice all their tics	A “comparison of tic assessment in adults, based on personal assessments vs. videotape analysis, showed that 90% still had tics despite frequent claims of their absence ^79^.” ^93^ “In clinical practice and in research it is common to observe tics in those who do not report any tic symptoms ^79^.” ^24^ In an epidemiological study, 27 of the 33 TS cases had never been diagnosed clinically with TS, and only one was being treated with a medication ^122^.
Parents don’t notice all their child’s tics Teachers don’t notice all their students’ tics	“Tics were coded as present if either a parent or a teacher reported them. … Not all subjects with preexisting tics were identified by both parents and teachers. Parents reported 18 of the 27 cases of tics, whereas teachers reported 14; only 5 cases of tics were identified by both parents and teachers.” ^49^ Teachers notice tics in fewer children than parents do [Table 2 in ref. 25, 34]. “Investigators who have relied strictly on historical data rather than on direct observation of symptoms have often been misled. For example, multiple recurrent tics were missed by parents in 21% of children being followed up in a longitudinal study. ^51^” [ 93, punctuation normalized] Direct expert examination and structured interviews of relatives of TS patients found over five times as many cases of TS and almost twice as many cases of CTD as did family history interviews alone ^123^. Three of 34 children or younger siblings of probands with TS had tics on exam though their parents had reported no tics ^50^.
Different observers notice tics in different children	In an overall sample of 867 children, parents identified tics in 71 children and teachers in 50 children; 23 were identified by parents and by teachers (κ=0.30, indicating only fair agreement). During a classroom visit an expert observed tics in 57 of the 867 children ^46^.
Physicians miss tics in clinical care	Of 58 children with TS in an epidemiological sample, 73% had had some kind of medical treatment, and of those, “more than 2 in 5 had received child psychiatric treatment, but only in 1 of 18 cases receiving such treatment had a diagnosis of tics of Tourette's disorder been considered. No child had received medication for tics or other neuropsychiatric disorder prior to the study” ^124^. A multiple-informant study in a school district estimated the prevalence of DSM-III-R TS as 3.0%, whereas only 0.05% of children in the area had been diagnosed by physicians ^42a^.
Observers are subject to confirmation bias	In a study of 867 children, in whom parents or teachers identified tics in 11.3%, 6.5% (58%) had tics on observation, but “most of [these] were not noted to have tics on a second observation period during which the observer was not told which children had screened positive by parents or teachers” ^46^.
Brief observations miss tics	Cubo showed that observers’ sensitivity increased from 33% to 58% as the length of observation increased from 1 to 3 hours [Table 2 in ref. 25].
More severe tics are probably noticed more consistently	Law & Schachar ^49^ note that in the two children who developed the most noticeable new tics, “symptoms were reported by both parents and teachers as soon as they developed.” Note however that one of the children already had other tics. Most of the tics in the Linazasoro *et al.* study were mild and had lasted only for a short period of time ^46^. Greater tic severity was significantly correlated with shorter time to diagnosis ^42^. Sixty (28%) of 214 children who screened positive for a lifetime history of DSM-IV TTD did not want to come for clinical examination ^34^.
Tic severity fluctuates dramatically over time, and also in response to setting, stress, and observation method	Tics fluctuate in severity over time. Tics are (usually) suppressible. Tics worsen with stress. Tics can worsen or improve with environmental cues unrelated to stress (even when people attribute no importance to the cues ^125^). People tend to tic more when unobserved ^126^. *The first item above is required by the Tourette Syndrome Study Group (TSSG) criteria for TS* ^117^ *, and the* *next three are mentioned in the Diagnostic Confidence Index, which identifies “classic” features of TS* ^127^.
Effective treatment can hide tics	In the Snider *et al.* study, two children had a clinical diagnosis of TS; tics were never seen in the child taking medication for tics, but were seen on four of eight visits in the unmedicated child ^31^. In that study, five children with a clinical diagnosis of motor tics ( *i.e.*, diagnosed as part of regular medical care, independent of the study) had no tics observed during the 8-month study, and three of these were on medication for tics.
Tics may be more common at different times of the year	Snider and colleagues reported tics in 10% of elementary school children when evaluated in November, 4–9% when evaluated in December, January or February, and 3–7% when observed between March and June ^31^. Clinicians commonly see more tic patients at the beginning of a school year.
Different observers differ in assessment of remission or change in severity	“In only 7 (25.9%) of the 27 subjects with preexisting tics did parents and teachers agree on the changes; in 2 (28.6%) of the 7 cases, parents reported a worsening of the tics, whereas teachers reported improvements” ^49^.
Over time, children, parents and teachers get used to mild tics and worry less about them	Parents often get used to tics over time and pay less attention to them or are less worried by them.
Retrospective dating of the first tic is difficult ^49, 68^	Often there is a long delay from the first appearance of a tic to when someone realizes it is a tic ^128^. Several children or parents noticed the current tic but did not realize that earlier movements or noises were also tics ^68^. Normal vagaries of memory. In a prospective study, nine (43%) of 21 children *not* diagnosed with tics at baseline, but diagnosed with tics at 4-year follow-up, reported at follow-up that tics had started before the baseline visit ^48^.
Nosological questions about brief remissions and recurrences	Discussed in various statements and position papers on DSM-5 or ICD11 ^24, 129– 132^, concluding that “The criterion that 3 months’ absence of symptoms disqualifies an individual from a diagnosis of TS is not in keeping with the waxing-and-waning course of this condition” ^133^. Nevertheless, having a tic for 2 months each year for 5 years seems quite different from having a tic for 60 months straight; in DSM-IV or DSM-IV-TR the intermittent case is diagnosed with 5 episodes of TTD, yet in DSM-5 both cases are diagnosed as TS.
Nosological questions about syndrome and severity	Older studies found tic disorders very rarely, likely in part because of identifying only severe, persistent cases. For example, a 1980 report on 4,258 school children concluded that childhood tic disorder affected only 0.23% of children—0.87% if they included single tics ^134^. However, the authors explicitly excluded “certain single, transient tics clearly driven by a focal irritation” (our translation). DSM-IV required marked distress or impairment in a life role to diagnose a tic disorder. This requirement dropped the rate of tic disorders by over 60% in a large population sample ^38^ and by over 80% in a school district study ^135^.
Classification difficulties with other complex repetitive behaviors	**Tics vs. compulsions.** Most tics and most compulsions can be distinguished clearly. Distinguishing features include cognitive and affective premonitory phenomena ( *i.e.*, obsessions) before most compulsions and somatic premonitory phenomena before many tics. However, some repetitive behaviors satisfy the definitions both of tics and of compulsions, and hence cannot be cleanly separated. For instance, the Y-BOCS symptom inventory includes touching and tapping ^136, 137^, while the YGTSS tic symptom checklist includes symmetry-provoked behaviors and “just right” phenomena ^138^. **Stereotypies.** DSM-5 draws attention to the following differences: compared to tics, stereotypies seen in autism or in Stereotypic Movement Disorder are involuntary, rhythmic, and stop “with distraction ( *e.g.*, name called or touched)”; they also tend to begin earlier in life (before age 3), last many seconds to minutes, lack a premonitory urge, and consist of a “constant repetitive fixed form and location” [ 12, under Stereotypic Movement Disorder]. Common “examples include repetitive hand waving/rotating, arm flapping, and finger wiggling.” Additionally, patients with Stereotypic Movement Disorder often find the movements rewarding or soothing and do not see them as a bothersome symptom or want them to be stopped ^139^; by contrast, seeking treatment appears on a consensus list of typical features of TS ^127^. **Normal behavior.** The YGTSS tic symptom checklist includes nail biting, knuckle popping and toe tapping, which are very common in children without tics ^138^. Trichotillomania and similar disorders may occur more often than chance in tic disorders, but are usually classified separately.
Non-tic behaviors that may be interpreted as tics by non-experts	Eyelid myoclonus. In 49 of 101 cases in which parents initially endorsed a question about one or more specific tics, a telephone interview revealed the symptoms not to be tics (false positives) ^41^. Examples included blinking or sniffing due to allergic rhinitis, making a face when annoyed, sighing, or vacant mouth opening. The authors of that report cite a 43% false positive rate in an older study with similar methods ^140^.
Tics that may not be recognized as tics by non- experts	Commonly: blinking, sneezing, coughing, throat clearing, jerking the head as if to throw hair out of the eyes. Complex tics are often misconstrued as intentional behavior. Mol Debes *et al.* reviewed the histories of 314 children with TS and found that diagnosis of TS occurred a median of 2.8 years after tic onset, and concluded that the diagnostic delay was primarily due to “a lack of knowledge about the normal course of TS among professionals and the public” ^128^.
Confusing diction	The word “tic” is also used (at least in English) for unrelated phenomena, including • tic douloureux (trigeminal neuralgia) • mannerisms (Merriam-Webster gives the example “Constantly playing with her hair is one of her more annoying tics” ^141^).

**Appendix 3, Table A1.** Calculation from the data of Snider *et al.* ^31^, assuming 8% as the estimated rate of chronic tic disorders (TS plus chronic motor tic disorder).

grade	N	no tics	motor tic(s)	motor tic(s) (%)	new vs. last year (min.)	new vs. 2+ yrs. ago	lifetime prevalence (min.)
**K**	83	66	17	20.5%			20.5%
**1 ^st^**	57	30	27	47.4%	13.4%	39.4%	47.4%
**2 ^nd^**	85	69	16	18.8%	0.0%	10.8%	47.4%
**3 ^rd^**	43	34	9	20.9%	1.1%	12.9%	60.3%
**4 ^th^**	98	69	29	29.6%	4.3%	21.6%	69.0%
**5 ^th^**	78	57	21	26.9%	0.0%	18.9%	79.2%
**6 ^th^**	109	93	16	14.7%	0.0%	6.7%	79.2%

**Appendix 3, Table A2.** Minimum lifetime prevalence of provisional tic disorder assuming various prevalence rates for chronic tic disorders and the results of Snider *et al* ^31^.

True rate of TS+CTD	Minimum lifetime prevalence of Provisional Tic Disorder by 6th grade
**1%**	93%
**2%**	91%
**3%**	89%
**4%**	87%
**5%**	85%
**6%**	83%
**7%**	81%
**8%**	79%
**9%**	77%
**10%**	75%
**11%**	73%
**12%**	71%

## Appendices

### Appendix 1. Nosological confusion

The literature is complicated by substantial confusion about the meaning of the phrase “transient tic disorder” (the nomenclature used most often in the existing literature). The word “transient” is taken to mean two different things. The Tourette Syndrome Study Group (TSSG) criteria ^117^ represent a usage in which “transient” means something that was present but has disappeared. The TSSG criteria for Transient Tic Disorder (TTD) require that tics began over a year ago, but lasted no more than 12 consecutive months; *i.e.*, the tics are now gone. Using the TSSG criteria, patients who are ticcing but whose tics started less than a year ago have “diagnosis deferred.” The ICD-10 criteria similarly require that “tics do not persist for longer than 12 months,” so that in the first year since onset one diagnoses “tic disorder, unspecified” ^118^.

Contrary to a common assumption ^24, 27, 119, 120^, DSM-IV-TR specified a *different* meaning of “transient” ^121^. The DSM-IV-TR criteria for TTD do *not* require disappearance of the tics, and the accompanying text clearly specifies: “Often, the diagnosis may change over time during the natural history of a Tic Disorder. For example, during the first months, a child may be diagnosed as having a Transient Tic Disorder. After a year, with further tics and longer duration, the diagnosis may become Tourette’s Disorder” ^121^. This usage—tics are transient until they prove themselves to be chronic—seems natural in the context of other DSM-IV-TR diagnoses such as Major Depressive Disorder that are based on current data but may need to be revised (e.g., if the patient later develops a manic episode, in which case the preceding depression is revealed to have been part of bipolar disorder, or if Alzheimer’s disease is diagnosed in life but autopsy reveals a different dementing illness). To deal with the patients left undiagnosed by this misunderstanding, some epidemiology studies claimed to use DSM-IV-TR criteria, but used “provisional” or “not otherwise specified” to describe patients with tics for less than a year, even though these patients in fact met DSM-IV-TR criteria for TTD.

Due to widespread dissatisfaction with the term Transient Tic Disorder, DSM-5 adopted the term “provisional tic disorder” (PTD) ^12, 24^. This diagnosis differs from DSM-IV-TR TTD not only in name and in the clarification that the diagnosis is intended for all children whose tics began less than a year ago, but also in two other substantive ways. First, PTD can be diagnosed within the first 4 weeks after tic onset. Second, and more consequentially, DSM-5 requires that any tic present more than 1 year after the first tic be diagnosed as TS or CTD (assuming the tics are not secondary to another illness or to a known toxin), disallowing the diagnosis of PTD for someone with current tics who had tics more than a year ago, regardless of any intervening asymptomatic period.

### Appendix 3. Minimum cumulative prevalence of tics

Excluding the relatively rare tic caused by drugs or a systemic illness, any child with a definite tic on even one examination can be diagnosed with DSM-5 PTD. If the tics continue beyond the 1-year anniversary of the first tic, the child still had PTD for the first year ^12^.

In one careful study, the highest cross-sectional prevalence of motor tics at any one observation was 9.6%, but since a number of children were seen to tic on one visit to the school but not another, 47% of first graders were observed to have a motor tic at some point during the year [Table 1 in ref. 31]. Admittedly, this rate may include some false positives, for instance nose wrinkling or sniffing that could better be explained by allergic rhinitis, since observers did not talk with the children or their parents. On the other hand, false negatives must also have occurred, as observers did not identify tics in several children with a clinical diagnosis of motor tics, and did not record vocal tics at all. A pediatric neurologist had trained the raters and performed reliability testing, and motor tics were counted only if they occurred three or more times in the same visit.

The relevant point for the present discussion comes from the fact that tics were observed in classrooms ranging from kindergarten to sixth grade, and collectively the results lead to an interesting conclusion about lifetime prevalence. For instance, 21% of third graders were also observed to tic. If the year the study was done was typical, then about 47% of the current third grade class would have shown tics if observed two years earlier, when *they* were in first grade. Even assuming a high true rate of chronic tic disorders (say, 8%), tics must have been new in at least 13% (= 21% − 8%) of the third graders *in addition to* the 47% who had tics in first grade. Applying this reasoning to the rest of Snider *et al.*’s data, by sixth grade 79% of students must have had at least one tic, and thus have met criteria for PTD at some point ( Table A1, below). This estimate for lifetime prevalence of PTD does not depend strongly on the assumed prevalence of chronic tic disorders ( Table A2), and is much higher than the cross-sectional prevalence rates reported in epidemiological studies or review articles.
