# Supplementary material for: Provisional Tic Disorder: What to tell parents when their child first starts ticcing
Source: F1000Res. 2016 Apr 18;5:696. [Version 1] doi: 10.12688/f1000research.8428.1 (PMC4850871; doi:10.12688/f1000research.8428.1)
Supplement: Peer review history

# Peer review: Referee response for version 1

**DOI:** 10.5256/f1000research.9072.r13530

**Published:** 2016-05-03

**Authors:** Jeremy Stern

**Affiliations:** 1 Department of Neurology, St. George's Hospital and Medical School, London, UK

The paper is a cogent and very useful exploration of an under-considered topic by critical review which challenges certain established clinical assumptions. The difficulties of estimating the prevalence of PTD and the risk of a newly ascertained case of tics progressing to a chronic disorder are discussed in detail, based on the literature. The inevitable conclusion of a need for (expensive) further prospective study is reached, and is an aspiration shared around the world. The appendices are particularly helpful, explaining problems of terminology, ascertainment of tics and the expected lifetime prevalence of PTD in the case of various prevalences of the other tic disorders.

​I very much agree with my fellow reviewer ( http://f1000research.com/articles/5-696/v1#referee-response-13477) that the clinical term "Tourette" may have outlived its usefulness, can be unhelpful to patients and clinicians and could be improved by now being subsumed into a concept of "tic spectrum disorder", notwithstanding our reverence for its birthplace or, more practically, the freshness of DSM V.

I have read this submission. I believe that I have an appropriate level of expertise to confirm that it is of an acceptable scientific standard.

# Peer review: Referee response for version 1

**DOI:** 10.5256/f1000research.9072.r13533

**Published:** 2016-04-28

**Authors:** Andrea Cavanna

**Affiliations:** 1 School of Life and Health Sciences, Aston Brain Centre, Wellcome Trust Laboratory for Magnetoencephalographic (MEG) studies, Aston University, Birmingham, UK

This is an interesting article on the nosographic category of provisional tic disorder. The authors provided a useful overview on the development of diagnostic categories for tic disorders which improve over time. The implications for clinical practice are particularly relevant.

I have read this submission. I believe that I have an appropriate level of expertise to confirm that it is of an acceptable scientific standard.

# Response

**Published:** 2016-04-29

**Authors:** Kevin J Black

**Affiliations:** Department of Psychiatry, Washington University in St Louis, USA

Thank you.

# Peer review: Referee response for version 1

**DOI:** 10.5256/f1000research.9072.r13477

**Published:** 2016-04-20

**Authors:** Andreas Hartmann

**Affiliations:** 1 Department of Neurology, Assistance Publique- Hopitaux de Paris, Paris, France

The authors must be congratulated for this thoughtful and exhaustive review on a difficult topic. I hope that their ongoing initiative ( www.NewTics.info) will provide answers to many of the questions raised in the manuscript.

My comments are few and as follows :

- The definition of tics varies but a reasonable argument has been made that they are not « abnormal » movements of vocalisations but rather normal or physiologic but occuring in an inappropriate context and/or in an inappropriate fashion. As a net result, one can of course speak of abnormal motor or vocal manifestations but as a movement disorder neurologist, I think it is important to stress that the semiology of tics does not give us clear diagnostic clues , as opposed to dystonia of parkinsonina tremor, for instance. Also, it is worth mentioning that tics can be (temporarily) suppressed, which is not the case in the vast majority of movement disorders. Finally, like it or not, « stereotyped » has been removed from DSM-5.
- I sympathize with the idea that early intervention, especially using behavioral therapies such as CBIT, may alter disease course, maybe through cerebral plasticity. However, there are no data to support this at present, even in confirmed TS cases. Moreover, CBIT (as opposed to ERP) depends on sensing premonitory urges, which are rarely present before the age of 10, and most PTD cases are likely to fall into this category. Finally, I believe that for patients with PTD, motivation and thus adherence for these kind of treatments will be low, except for severe cases. Thus, the therapeutic angle appears slightly artificial to me.
- Rather, and this is the main question raised in the manuscript and by parents of children with PTD, is the issue of individual prognosis, and especially conversion of of PTD into TS. The problem, at least in France, is that tics are considered benign, and TS almost malignant. In other words, the terminology we use is paramount. My personal concern is not so much whether one or several tics will persist for more than a year but how damaging they are. This brings us to the talmudic discussion on the utility of DSM criteria and even more on what Tourette syndrome means or not (the authors point out quite rightly that in current clinical pratice, we are usually light years away from the Marquise de Dampierre). If I had a wish, I would get rid of the term « Tourette syndrome » and switch to tic spectrum disorder (or something like that), although I concede this may be a mushy term. However, I don’t expect to see this happen in my lifetime.

I have read this submission. I believe that I have an appropriate level of expertise to confirm that it is of an acceptable scientific standard.

# Response

**Published:** 2016-04-29

**Authors:** Kevin J Black

**Affiliations:** Department of Psychiatry, Washington University in St Louis, USA

We appreciate Prof. Hartmann's thoughtful comments.
